# Video Clip Extraction From Fetal Ultrasound Scans Using Artificial Intelligence to Allow Remote Second Expert Review for Congenital Heart Disease

**DOI:** 10.1002/pd.6757

**Published:** 2025-02-06

**Authors:** Thomas G. Day, Lorenzo Venturini, Samuel F. Budd, Alfonso Farruggia, Robert Wright, Jackie Matthew, Vita Zidere, Trisha Vigneswaran, Ilaria Bo, Alex Savis, Jo Wolfenden, John Simpson, Jo Hajnal, Bernhard Kainz, Reza Razavi

**Affiliations:** ^1^ School of Biomedical Engineering and Imaging Sciences King’s College London London UK; ^2^ Fetal Cardiology Unit Department of Congenital Heart Disease Evelina London Children’s Healthcare Guy’s and St Thomas’ NHS Foundation Trust London UK; ^3^ Fetal Cardiology Unit Royal Brompton and Harefield Hospitals Guy’s and St Thomas’ NHS Foundation Trust London UK; ^4^ Fetal Cardiology Unit Great Ormond Street Hospital for Children NHS Foundation Trust London UK; ^5^ Department of Computing Imperial College London London UK; ^6^ Image Data Exploration and Analysis Lab Friedrich‐Alexander‐Universitat Erlangen‐Nurnberg Erlangen Germany

## Abstract

**Objective:**

To use artificial intelligence (AI) to automatically extract video clips of the fetal heart from a stream of ultrasound video, and to assess the performance of these when used for remote second review.

**Methods:**

Using a dataset from a previous clinical trial of AI to assist in fetal ultrasound scanning, AI was used to automatically extract video clips of the fetal heart from ultrasound scans of 48 fetuses in which the diagnosis was known: 24 normal and 24 with congenital heart disease (CHD). These, and manually still saved images, were shown in a random order to expert clinicians, who were asked to detect cardiac abnormalities.

**Results:**

The initial manual scan had a sensitivity of 0.792 and specificity of 0.917 for detecting CHD in this cohort. The addition of second review improved the sensitivity to 0.975 using video clips, which was significantly higher than using still images (0.892, *p* = 0.002). There was a significant drop in specificity to 0.767 and 0.833 (*p* < 0.001) for the video and still method, respectively, which were statistically similar to each other (*p* = 0.117). The median review time was 1.0 min (IQR 0.71) for the still images, and 3.75 min (IQR 3.12) for the AI‐generated video clips.

**Conclusion:**

AI can be used to automatically extract fetal cardiac video clips, and these can be used for remote second review to improve detection rates. Video clips are superior to still images, but both methods result in a significant drop in specificity.

**Summary**

- What is already known about this topic?
  ◦ Many imaging‐based screening programmes employ a review of the imaging data by a second clinician, as this is known to improve diagnostic performance.
  ◦ In fetal ultrasound, a single review at the time of scan is the standard practice.
  ◦ Current fetal anomaly screening programmes are failing to detect a significant proportion of serious fetal anomalies, particularly congenital heart disease.
- What does this study add?
  ◦ Artificial intelligence can be used to automatically extract video clips containing the standard cardiac views from a continuous stream of ultrasound video data, without a change in sonographer workflow.
  ◦ These clips can be used to facilitate a second review by another clinician.
  ◦ Video clips are superior to manually saved still images for second review.
  ◦ The second review results in an improvement in screening sensitivity, but at some cost of specificity.

## 1 Background

The Fetal Anomaly Screening Program (FASP) offers a screening ultrasound scan to all pregnant patients in the UK at between 18^+0^ and 21^+6^ weeks’ gestation, with similar schemes in most economically‐developed countries [1]. The purpose of these scans is to detect major fetal anomalies so that antenatal and postnatal care can be optimized, including parental choice on whether they want to continue with the pregnancy. Unfortunately, these screening programmes are falling well short of universal detection. As an example, for congenital heart disease (both the most common and most deadly group of fetal malformations), the antenatal detection rates in the UK are around 54%, with wide regional and international variation [2, 3, 4].

For many imaging‐based medical screening programmes (e.g. screening mammography for breast cancer), images are reviewed by two expert clinicians, a method termed double reading [5]. This practice has been shown to improve the sensitivity and therefore detection rates [5]. However, with fetal ultrasound screening, a single reading is the norm. In the UK, sonographers are only required to save and archive a very limited selection of still images, with no requirement for storage of video clips or second review [1].

Some researchers have investigated the feasibility of remote telemedicine input by a specialist for fetal ultrasound, for example, a fetal cardiologist reviewing scans that were deemed high risk for congenital heart disease, or live review of first trimester scanning [6, 7]. Despite promising initial findings, these models have not become widespread in the UK. The barrier to adoption of these telemedicine service models is that either (a) the standard saved still images are used, which have been shown to be inferior for diagnosis compared to video clips [8], (b) sonographers have to alter their practice to save video clips rather than still images, or (c) the entire ultrasound scan has to be reviewed, which would be extremely time‐consuming and hence prohibitively expensive.

We recently undertook a randomized controlled trial of an artificial intelligence (AI) based tool to assist sonographers when performing the routine screening fetal anomaly ultrasound scan [9]. The tool performed automated fetal biometry, and also automatically saved images that corresponded to a number of standard image planes. We found that use of the tool significantly reduced both scan duration and sonographer cognitive load, but did not significantly change detection rates of fetal congenital heart disease [9]. As the AI tool automatically labeled every frame of the ultrasound video as a standard plane (or background), it was possible to use these labeled data to reconstruct video clips. During the trial, each participant also underwent a standard manual ultrasound scan, with archiving of standard still images.

The aim of the present study was to investigate the feasibility of using these AI‐acquired video clips to allow a remote expert second review of the ultrasound scans, and to compare the diagnostic performance of this with the remote review of the standard saved still images, and the diagnostic performance of the original manual scan. We focused on congenital heart disease as this is the most common group of congenital abnormalities, so is the most obvious target for any effort to improve antenatal detection rates.

The primary outcome was the diagnostic performance (sensitivity and specificity) of the second review for AI‐acquired video clips versus manually‐acquired still images. The secondary outcome was the time taken for the secondary review using both methods.

## 2 Methods

A randomized controlled trial of AI‐assisted versus standard manual fetal anomaly screening ultrasound was performed in a large London teaching hospital, and is fully described in a previous publication [9]. This trial involved 78 pregnant participants (carrying either a healthy fetus or a fetus with known CHD) undergoing two ultrasound scans, once with each method.

GE Voluson Expert 22 machines were used for all scans. Inclusion criteria for the original trial were either diagnosis of fetal CHD between 12^+0^ and 27^+6^ weeks gestation (for the CHD group) or confirmation of normal fetal cardiac anatomy between 18^+0^ and 27^+6^ weeks gestation (for the normal group). Exclusion criteria for pregnant participants were: being under 18 years of age, multiple pregnancy, any plan for termination of pregnancy, any known fetal extracardiac structural abnormality at the time of recruitment, any known fetal genetic abnormality, refusal of consent, or insufficient English language skills to provide informed consent. All sonographers were employed by NHS trusts and undertook routine fetal anomaly ultrasound scans as part of their daily work.

During the AI‐assisted scan, the novel AI tool was used. This takes as input every frame of the ultrasound scan video obtained by the sonographer, and automatically classified each frame into one of 13 standard image plane labels, or background. This included five cardiac planes (abdominal situs view, four‐chamber view (4CH), left ventricular outflow tract view (LVOT), right ventricular outflow tract view (RVOT), and three vessel tracheal views (3VT)). This step was performed by the SonoNEXT model and is fully described in a separate publication [9], but briefly this model is a type of convolutional neural network, taking as input a single ultrasound image frame, and producing a prediction of the most likely standard image plane label. Each image was also automatically assigned a quality score. Again, full information on the quality scoring model is available in our previous publication [9], but briefly this is also a convolutional neural network, taking as input a single image and producing an output that is a predicted quality score on a continuous scale, also taking into account image diversity (to avoid multiple very similar images being selected as the best quality). Details of the development and performance of the AI models are described in the previous publication [9]. The AI models were run in real time during the scans on a computer equipped with a graphical processing unit (Boxer‐8641AI, Aaeon Technology Inc., Taipei, Taiwan), which was connected to the ultrasound machine via a high‐definition multimedia interface (HDMI) connection. Information on AI model output was displayed to the sonographer via a tablet (iPad Air Fifth Generation, Apple Inc. Cupertino, United States of America). The tablet was connected to the computer via a Wi‐Fi connection.

During the manual scan, the sonographers were asked to save a single still image for each of the same 13 standard planes, including the same five cardiac views. These images were selected and saved during the scan by the scanning sonographers, as usual clinical procedure.

**FIGURE 1:**
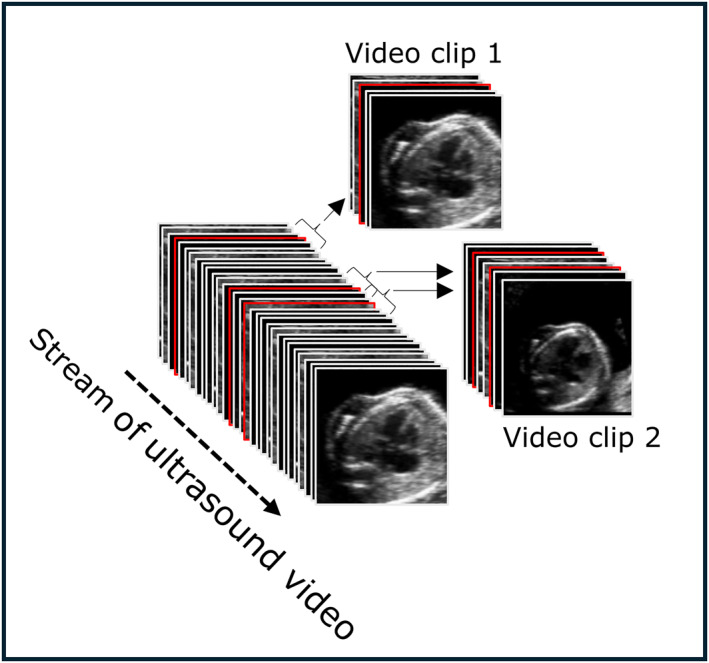
Extraction of video clips from a stream of ultrasound video. At the end of the scan, the nine highest quality images were automatically selected for each cardiac plane (shown with red border, with only three demonstrated in this figure for clarity). The period 2.5 s before, and 2.5 seconds after that frame were taken to give a 5 s video clip (e.g. video clip 1). If the time periods overlapped between different highest quality frames, then the clips were merged into a single longer clip (as shown with video clip 2).

For each AI‐assisted scan, the nine highest quality images for each of the five cardiac planes were selected. Using each of these, a five‐second video clip was created with the high‐quality frame as the central frame (i.e. the 2.5 s period prior and the 2.5 s after that given frame were taken to produce a 5 s video clip). If any clips overlapped temporally, they were merged into a single clip. This process is demonstrated in Figure 1. This led to the creation of a dataset of up to nine video clips per cardiac plane, per patient, as well as the manually saved still cardiac images for the same patients. Supporting Information S1: Figure S1 and Movie S1 show an example of a manually saved image and an automatically extracted video clip, respectively.

Twenty four scans were available for fetuses with CHD. Using a random number generator (randomizer.org), 24 patients with normal fetal hearts were selected from the study population to form a cohort of 48 patients, each with AI‐acquired video clips of the fetal heart and with manually saved still images of the fetal heart. The AI‐acquired video clips and manually acquired still images were treated as separate scans, giving a total of 96 scans for review. Each scan was given a unique study ID. We did not include all the available scan data from the original trial for pragmatic reasons, so that the reviewers could review the imaging dataset in a reasonable amount of time.

**FIGURE 2:**
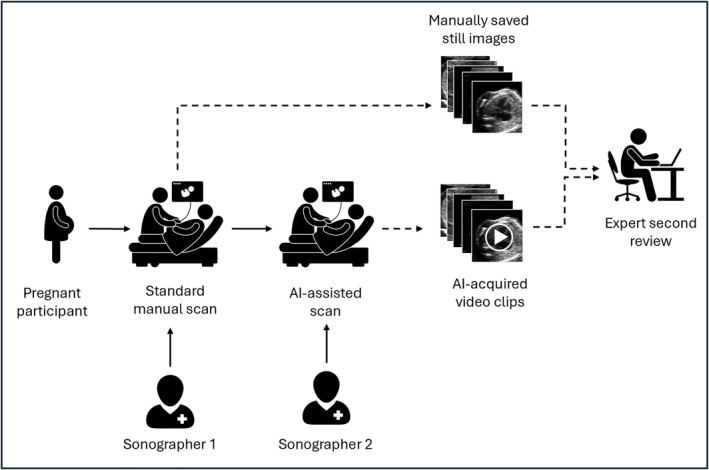
Study design diagram. Solid arrows: flow of participants; dashed arrows: flow of image data.

Five experts in fetal cardiology (not involved in scanning of the trial participants) were recruited from three hospitals (three consultants and two cardiac physiologists). The order of the scans was randomized using a random number generator (randomizer.org), stratified such that the two scans from the same patient did not appear in the same 50% of the scan order, to minimize memory effects. The fetal cardiac experts were shown each scan in a random sequence, and asked to image that they had been asked to undertake a remote secondary review of the scan imaging data. For each scan, they were asked to choose a single outcome from one of three possibilities: a) normal, no need for specialized cardiac review; b) abnormal, needs to attend fetal cardiac clinic; or c) uncertain, images insufficient for decision. The time taken to review the imaging data for each patient was recorded using a standard stopwatch. The time taken for the entire manual scan (and hence the time that would be required to review the entire video of the scan) was extracted from the original trial data. Figure 2 outlines the design of the study.

The study aimed to mimic a clinical scenario in which experts would perform a second review of the ultrasound scan imaging data, flagging patients who in their opinion required further assessment. As there was an intermediate “uncertain” option, the responses of the expert reviewers could be calibrated to be either highly sensitive (whereby uncertain cases were treated as screen positive and flagged as abnormal), or highly specific (whereby uncertain cases were treated as screen negative and not flagged as abnormal). The results were analyzed both considering the second review in isolation, and when combined with the outcome from the initial manual scan (i.e. a case was flagged as abnormal if either the initial manual scan or the subsequent second review identified the abnormality, as would be the likely real‐world clinical pathway).

The primary outcome measures of sensitivity and specificity were compared statistically using McNemar’s test for paired proportions. The secondary outcome measure of review duration was compared using the Wilcoxon signed ranks test. Ninety‐five percent confidence intervals were calculated using the exact Clopper‐Pearson method.

## 3 Results

Baseline data on pregnant participants are shown in Table 1. The 96 scans (imaging data from 48 patients as either still images or as AI‐acquired video clips) were analyzed by five expert reviewers, giving 480 decisions.

**TABLE 1:** Baseline characteristics of pregnant participants. Data are *n* (%) or mean (SD).

	CHD affected group (*n* = 24)	CHD unaffected group (*n* = 24)
Maternal age (years)	31.9 (5.3)	31.4 (5.5)
Gestational age (weeks)	24.9 (1.7)	23.8 (1.3)
Body mass index (kg/m^2^)	26.9 (5.1)	27.8 (5.6)
Ethnicity		
White	19 (79.2%)	24 (100%)
Black or mixed black	2 (8.3%)	0 (0%)
Asian or mixed Asian	1 (4.2%)	0 (0%)
Any other	2 (8.3%)	0 (0%)

Table 2 shows the breakdown of diagnoses in the CHD group. Table 3 shows the number of studies that included at least one video clip or still image per image plane. All video clip datasets included at least one video for every cardiac plane, and for the still image method between 85.4% and 97.9% of studies had at least one image for review, depending on plane (with images missing either because the sonographer felt the plane was technically unfeasible during the scan, or because of operator error).

**TABLE 2:** Fetal cardiac diagnoses in the congenital heart disease group.

Fetal CHD lesion	Number (%)
Right aortic arch	8 (33.3%)
Transposition of the great arteries	3 (12.5%)
Tetralogy of fallot	2 (8.3%)
Double aortic arch	3 (12.5%)
Atrioventricular septal defect	2 (8.3%)
Bilateral superior venae cavae	2 (8.3%)
Hypoplastic left heart syndrome	1 (4.2%)
Double‐outlet right ventricle	1 (4.2%)
Hypoplastic aortic arch	1 (4.2%)
Pulmonary stenosis, right aortic arch, interrupted inferior vena cava	1 (4.2%)
**Total**	**24 (100%)**

**TABLE 3:** Number of studies with at least one still image or video clip per image plane.

	Abdo	4CH	LVOT	RVOT	3VT
Manually acquired still images	47 (97.9%)	47 (97.9%)	46 (95.8%)	43 (89.6%)	41 (85.4%)
AI‐acquired video clips	48 (100%)	48 (100%)	48 (100%)	48 (100%)	48 (100%)

Abbreviations: Abdo, abdominal situs view; LVOT, left ventricular outflow tract view; RVOT, right ventricular outflow tract view; 4CH, four chamber view, 3VT: three vessel tracheal view.

**FIGURE 3:**
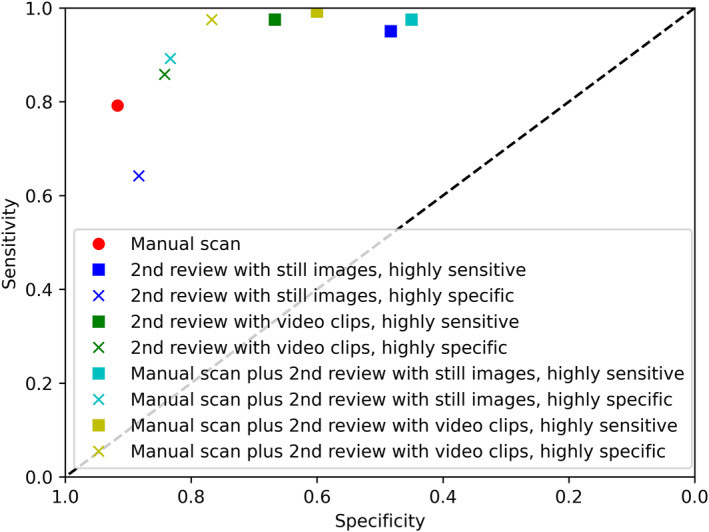
Diagnostic performance of the original manual scan, and the various second review methods.

Table 4 and Figure 3 show the diagnostic performance of each method considered independently, as well as the performance of the standard manual scan. The sensitivity and specificity of the original manual scan in the clinical trial for these patients were 0.792 and 0.917 respectively. The highly sensitive calibration of both second review methods (i.e. treating “uncertain” findings as screen positive requiring onward referral) achieved a superior sensitivity compared to the manual scan (0.950 for still images, 0.975 for video clips, *p* < 0.001 for both), but at the expense of inferior specificity (0.483 for still images, 0.667 for video clips, *p* < 0.001 for both). However, the specificity of the video clip method was superior to that of the still image method (*p* = 0.003). The highly specific calibration (i.e. treating “uncertain” findings as screen negative, not requiring onward referral) had inferior sensitivity and similar specificity to the manual scan for the still image review methods, and both similar sensitivity and specificity for the video clip review methods.

**TABLE 4:** Diagnostic performance of the standard manual scan and second review methods considered independently.

	Sensitivity (95% CI)	*p* value^a^	*p* value^b^	Specificity (95% CI)	*p* value^a^	*p* value^b^
Manual scan	0.792 (0.708–0.860)	—	—	0.917 (0.862–0.959)	—	—
2^nd^ review with still images						
*Highly sensitive*	0.950 (0.894–0.981)	**< 0.001**	—	0.483 (0.391–0.576)	**< 0.001**	—
*Highly specific*	0.642 (0.549–0.727)	**0.005**	—	0.883 (0.812–0.935)	0.317	—
2^nd^ review with video clips						
*Highly sensitive*	0.975 (0.929–0.995)	**< 0.001**	0.317	0.667 (0.575–0.750)	**< 0.001**	**0.003**
*Highly specific*	0.858 (0.783–0.915)	0.182	**< 0.001**	0.842 (0.764–0.902)	0.083	0.369

*Note:* P values considered statistically significant (<0.05) are in bold.

^a^ *p* value compared with manual scan.

^b^ *p* value compared with still image second review.

Next, the results were analyzed with the findings of the original manual scan combined with the second review, i.e. the scan was deemed screen positive if either the original scan or second review was positive, and only negative if both were negative. This likely represents how a second review system would work in clinical practice, whereby the second review would take place non‐contemporaneously, and would only be of scans initially felt to be normal. These findings are shown in Table 5 and Figure 3. The addition of a second review improved the sensitivity using either method, regardless of calibration (sensitivity 0.975 and 0.892 for the still images method highly sensitive and highly specific calibration, respectively; 0.992 and 0.975 for the video clip method). However, there was a significant deterioration in the specificity (0.450 and 0.883 for the still images method highly sensitive and highly specific calibration, respectively; 0.6 and 0.767 for the video clip method). The highly specific calibration of the video clip method resulted in a superior sensitivity to the still image method, with a specificity that was not statistically different.

**TABLE 5:** Diagnostic performance of the standard manual scan and second review methods combined with the manual scan.

	Sensitivity (95% CI)	*p* value^a^	*p* value^b^	Specificity (95% CI)	*p* value^a^	*p* value^b^
Manual scan	0.792 (0.708–0.860)	—	—	0.917 (0.862–0.959)	—	—
2^nd^ review with still images combined with manual scan						
*Highly sensitive*	0.975	**< 0.001**	—	0.450	**< 0.001**	—
*Highly specific*	0.892	**< 0.001**	—	0.833	**< 0.001**	—
2^nd^ review with video clips combined with manual scan						
*Highly sensitive*	0.992	**< 0.001**	0.317	0.600	**< 0.001**	**0.009**
*Highly specific*	0.975	**< 0.001**	**0.002**	0.767	**< 0.001**	0.117

*Note:* *P* values considered statistically significant (<0.05) are in bold.

^a^ *p* value compared with manual scan.

^b^ *p* value compared with still image second review.

The secondary outcome measure was the time taken for the second review. For the still images, the median review time was 1.0 min (IQR 0.71), and for the video clips, the median review time was 3.75 min (IQR 3.12), *p* < 0.001. The median duration of the full scan video (and hence, the estimated time to review the entire scan) were 20.2 min (IQR 8.82).

## 4 Discussion

We have investigated the possibility of using AI to facilitate efficient second review of fetal anomaly screening ultrasound scans. The use of the automatically generated video clips for second review showed superior performance compared to when the manually acquired still images were used for this purpose. We examined both the use of second review alone, and in conjunction with the initial diagnosis from the scanning sonographer. This approach is likely representative of how such a system could work in real clinical practice, with a fetus flagged as abnormal if either the primary scanning sonographer or a second reviewer identified a problem. The second review could take place remotely and non‐contemporaneously, in a variety of different configurations.

We found that when AI‐acquired video clips were used for second review, 89.2%–97.5% of cardiac anomalies were detected when used alone and 97.5%–99.2% when used in conjunction with the findings from the original scan. This is a significant improvement compared to the sensitivity of the original scan of 0.792, meaning that with this proposed novel workflow, more infants could be diagnosed with CHD antenatally, rather than being diagnosed after birth. In addition, we have shown that this second review using video clips takes on average only 3.75 min per patient, which although longer than using still images for review, is still much shorter than reviewing the entire ultrasound scan video. In addition, although not part of this present study, the original trial demonstrated that using the AI tools resulted in a significant reduction in scan duration, which will at least in part compensate for the addition time required for the second review of video clips [9].

However, this does come at the cost of reduced specificity. Combining second review of video clips with the original scan resulted in a specificity of 0.6–0.767, significantly worse than the specificity of the initial manual scan. Whilst some drop in specificity when introducing a second review workflow is inevitable (the specificity can only remain the same, or get worse, unless the second reviewer has the power to overrule a screen positive finding from the initial scan), these specificity figures are probably too low for current clinical use. If this level of specificity was extrapolated to a national screening program, this would result in a very large number of false positive results, probably beyond the capability of current screening services to offer a repeat or specialist scan [10].

However, this is an initial feasibility study, with no specific training provided to the expert reviewers taking part in the study. With a program of training, it may be possible to strike an acceptable balance between improving detection rates whilst keeping the false positive results to within an acceptable limit. We also have not explored in this study the use of AI to automate the detection of disease. Other studies have shown promising results in this area, and we are planning future research projects to investigate how automated AI disease detection could be combined with human expert second review, to optimize both sensitivity and specificity [10, 11, 12, 13].

There would also be a cost implication of employing a second review of scans. Previous studies have suggested that such telemedicine services for fetal ultrasound may be cost‐effective [6], but we have not assessed this in the present study. Improving rates of antenatal diagnosis would come with significant cost savings, but whether this is enough to justify a more expensive workflow would require a full health economic study, which is currently being planned. Those working as second reviewers would also require specific training in order to undertake this role, perhaps combined with approaches (such as artificially enriching the studies for review with known true positive cases) to ensure attention is maintained and sensitivity optimized.

The main limitation of the present study is the small sample size of patients and the number of expert second reviewers. Our findings will require confirmation using a much larger sample size, and we have plans to undertake a trial that is both larger, and uses real‐world clinical imaging data. It would be more representative to replicate the real‐world prevalence of CHD, i.e. around 1%, but this would require a huge increase in overall sample size to be able to include enough pathological examples. Such a trial would likely use AI models that have been retrained using significantly larger datasets, as these become available to us, with potentially superior model performance.

Secondly, although the initial clinical trial that formed the basis of this work aimed to simulate a clinical environment, these ultrasound scans were taken in addition to the standard clinical pathway, and thus may not be fully representative of the intended clinical population (both in terms of the pregnant participants who took part, and the volunteer sonographers). Only a single model of ultrasound machine was used, and further studies will be required across different centers and types of ultrasound machine, to ensure that these data are generalizable. Finally, the expert second reviewers were all specialists in the fetal heart. The use of such experts may not be realistic if such a workflow is rolled out nationally, as these clinical services are small and highly specialized. Further work should also investigate what level of performance can be achieved with other, less specialized workforces.

In conclusion, our results indicate that using AI to automatically extract ultrasound video clips of the fetal heart can be an effective way of facilitating expert second reviews. This results in an improvement in detection rates for CHD, superior to when manually acquired still images are used, but both methods result in a deterioration in specificity. Further work to integrate AI disease detection is ongoing, and may be a way of achieving an improvement in CHD detection, without an unacceptable increase in false positive diagnoses.

## Ethics Statement

This study was approved by the London Dulwich Research Ethics Committee, reference 22/LO/0163.

## Consent

Written informed consent was obtained from all participants in the trial from where the data were derived.

## Conflicts of Interest

TD, LV, SB, AF, RW, JM, JH, BK and RR are co‐founders and shareholders of Fraiya Ltd, a university/NHS spin‐out company aiming to commercialise the use of artificial intelligence in obstetric ultrasound. The imaging data used in this project were acquired using an early prototype of the planned commercial product.

## Supporting information

Supporting Information S1

Movie S1

## Data Availability

Patient‐level imaging data from the trial, and the imaging data used to train the AI models are not available for sharing due to ethical restrictions. The data that support the findings of this study are available from the corresponding author upon reasonable request.
