# Supplementary material for: Video Clip Extraction From Fetal Ultrasound Scans Using Artificial Intelligence to Allow Remote Second Expert Review for Congenital Heart Disease
Source: Prenat Diagn. 2025 Feb 6;45(4):531–8. doi: 10.1002/pd.6757 (PMC11987776; doi:10.1002/pd.6757)
Supplement: Supplementary file 1 — Supporting Information S1 [file PD-45-531-s001.docx]

Supplementary figure 1: an example of a manually saved four chamber view. In this the example is known to have an atrioventricular septal defect.


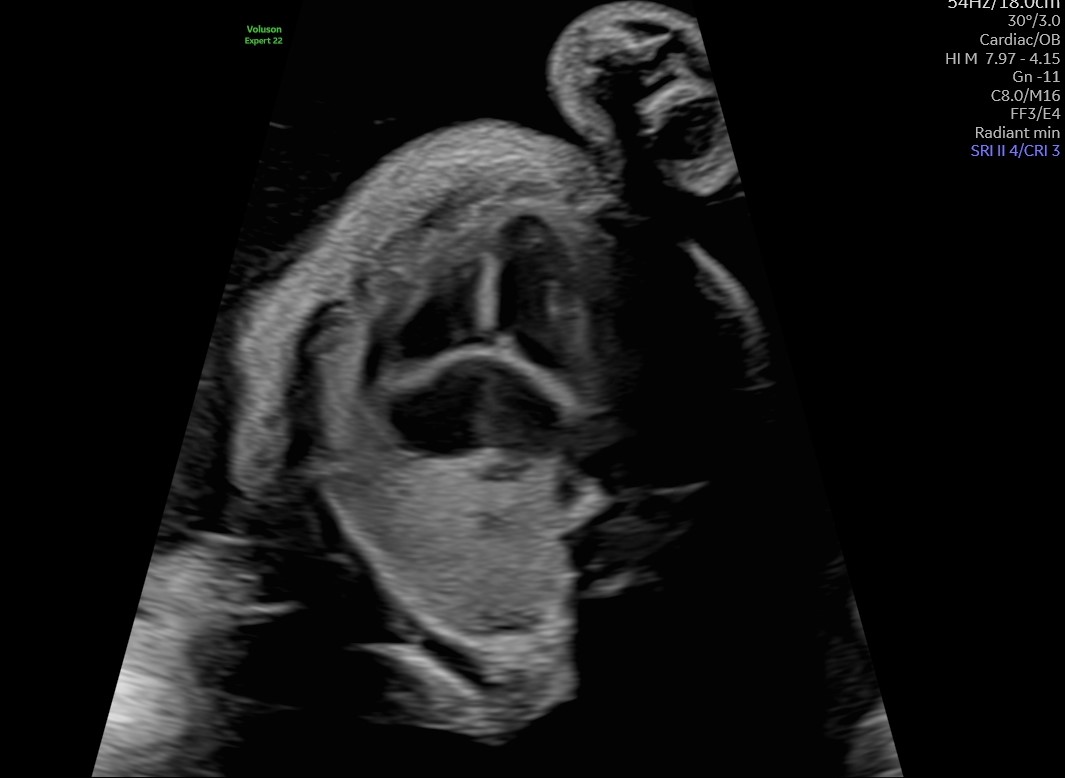


Supplementary video 1: an example of an automatically extracted video, with the central frame of the video being classified as a four chamber view. This is the same case as in supplementary figure 1, with the fetus known to have an atrioventricular septal defect.
